# Synaptamide Improves Cognitive Functions and Neuronal Plasticity in Neuropathic Pain

**DOI:** 10.3390/ijms222312779

**Published:** 2021-11-26

**Authors:** Anna Tyrtyshnaia, Anatoly Bondar, Sophia Konovalova, Igor Manzhulo

**Affiliations:** A.V. Zhirmunsky National Scientific Center of Marine Biology, Far Eastern Branch, Russian Academy of Sciences, Vladivostok 690041, Russia; bondar.av@dvfu.ru (A.B.); sofanasrew@gmail.com (S.K.); i-manzhulo@bk.ru (I.M.)

**Keywords:** synaptamide, N-docosahexaenoylethanolamine, neuropathic pain, spared nerve injury, hippocampus, neuroinflammation

## Abstract

Neuropathic pain arises from damage or dysfunction of the peripheral or central nervous system and manifests itself in a wide variety of sensory symptoms and cognitive disorders. Many studies demonstrate the role of neuropathic pain-induced neuroinflammation in behavioral disorders. For effective neuropathic pain treatment, an integrative approach is required, which simultaneously affects several links of pathogenesis. One promising candidate for this role is synaptamide (N-docosahexaenoylethanolamine), which is an endogenous metabolite of docosahexaenoic acid. In this study, we investigated the activity of synaptamide on mice behavior and hippocampal plasticity in neuropathic pain induced by spared nerve injury (SNI). We found a beneficial effect of synaptamide on the thermal allodynia and mechanical hyperalgesia dynamics. Synaptamide prevented working and long-term memory impairment. These results are probably based on the supportive effect of synaptamide on SNI-impaired hippocampal plasticity. Nerve ligation caused microglia activation predominantly in the contralateral hippocampus, while synaptamide inhibited this effect. The treatment reversed dendritic tree degeneration, dendritic spines density reduction on CA1-pyramidal neurons, neurogenesis deterioration, and hippocampal long-term potentiation (LTP) impairment. In addition, synaptamide inhibits changes in the glutamatergic receptor expression. Thus, synaptamide has a beneficial effect on hippocampal functioning, including synaptic plasticity and hippocampus-dependent cognitive processes in neuropathic pain.

## 1. Introduction

Neuropathic pain is a condition resulting from damage or dysfunction of the peripheral or central somatosensory system, rather than stimulation of pain receptors [1]. Difficulties in the treatment of patients with neuropathic pain are due to the heterogeneity of the etiology, symptoms, and underlying mechanisms of this condition. Difficulties often arise in determining the origin and exact location of the lesion or in elucidating the relationship between the deterioration of the patient’s condition and the neuropathic pain present. Causes of neuropathic pain include traumas of the central and peripheral nervous system, and various diseases, including multiple sclerosis, diabetes, herpesvirus infection, etc. [2]. As a rule, neuropathic pain is accompanied by sensory symptoms such as causalgia-intense, persistent burning pain, often of a lancinating nature. Causalgia is often associated with allodynia, a condition in which pain is caused by stimuli that usually do not cause pain, and hyperalgesia, which is characterized by increased pain when exposed to a stimulus that usually causes minor pain [3]. Among the mechanisms for neuropathic pain development, peripheral and central ones are distinguished. Peripheral mechanisms include direct stimulation of sensory nerves, peripheral sensitization of nociceptors by inflammatory mediators and biologically active substances, abnormal ectopic spontaneous activity of damaged nerves, increased activity of adrenergic receptors on axonal membranes, etc. [4]. Central mechanisms imply the participation of both the spinal and supraspinal centers in the generation, processing, and transmission of the pain signal [5].

Neuroplastic processes are considered to occur in the central nervous system in neuropathic pain and cause an imbalance between excitatory and inhibitory processes [6,7]. These processes are usually described by the general term "central sensitization" [8]. The involvement of supraspinal centers in the processing and transmission of pain signals makes neuropathic pain an even more complex phenomenon, including sensory-discriminatory, affective-motivational, and cognitive-evaluative components [9]. Moreover, the lateral pain system, which passes through the lateral nuclei of the thalamus into the primary and secondary somatosensory cortex, is involved in the sensory-discriminatory aspects of pain processing, providing the ability to analyze the intensity, duration, and location of pain stimulus [10]. The medial system, which passes through the medial nuclei of the thalamus into the prefrontal and anterior cingulate cortex, is responsible for the affective-motivational component, that is, it gives an idea of how unpleasant it is to feel pain [11]. The cognitive-evaluative axis of pain is probably associated with higher brain centers responsible for attention and memory [12]. It is well documented that neuropathic pain causes not only sensory symptoms but also cognitive and affective dysfunctions [6]. This confirms the involvement of higher supraspinal centers in neuropathic pain pathogenesis. Based on the data presented in the review [9], we can conclude that the hippocampus may be involved in various aspects of pain processing. Several studies demonstrate hippocampus-dependent memory impairment in neuropathic pain [13,14,15,16,17,18,19]. With chronicity, neuropathic pain may cause memory impairment, anxiety, depression, insomnia, etc. [20]. Many of the above studies demonstrate the role of neuroinflammation, including microglial activation and proinflammatory cytokine production, in the development of behavioral disorders. Thus, for effective neuropathic pain treatment, an integrated approach that simultaneously affects several links of pathogenesis is required. One of the promising candidates for this role may be N-docosahexaenoylethanolamine (synaptamide), which is an endogenous metabolite of docosahexaenoic acid. Synaptamide is synthesized in the body of mammals and plays an important role in many processes, including nervous system functioning. In vitro experiments have shown that synaptamide stimulates neuronal differentiation of neural stem cells [21], promotes neurite growth [22,23], stimulates synapse formation in cultured neurons [24,25], and suppresses neuroinflammation [26]. New evidence suggests nociceptive effects of synaptamide in acute pain [27]. In the present study, we focused on changes in glial, neuronal, and synaptic plasticity, which are the physiological and morphological substrates of memory changes, in neuropathic pain and synaptamide treatment.

## 2. Results

### 2.1. Synaptamide Improves Behavioral Parameters in Neuropathic Pain

**Figure 1:**
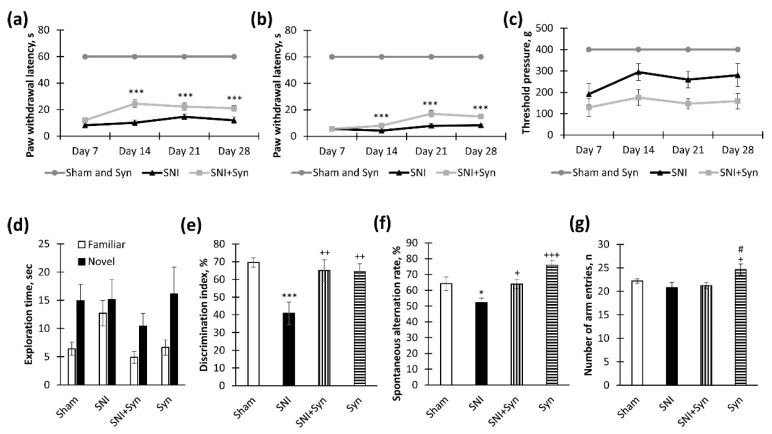
Effects of neuropathic pain and synaptamide therapy on behavior. (**a**) Hot allodynia dynamics: moment of hind paw lifting over hot plate (+48 °C) during 1-min observation, mean SEM, *n* = 20 (number of animals), *** *p* < 0.001. (**b**) Cold allodynia dynamics: the moment of hind paw lifting over the cold plate (+4 °C) during a 1-minute observation, mean ± standard error of the mean (SEM), *n* = 20 (number of animals), *** *p* < 0.001. (**c**) Mechanical hyperalgesia dynamics: the moment of toe flexor reaction following paw compression, mean SEM, *n* = 20 (number of animals),*** *p* < 0.001, ** *p* < 0.01, * *p* < 0.05 (**d**) Exploration time in novel objects recognition test, mean ± SEM, *n* = 20 (number of animals), * *p* < 0.05. (**e**) Discrimination index in novel object recognition test, mean ± SEM, *n* = 20 (number of animals), *** *p* < 0.001 (compared to “Sham”), ^++^ *p* < 0.01 (compared to “SNI”). (**f**) Spontaneous alternation rate in Y-maze, mean ± SEM, *n* = 20 (number of animals), * *p* < 0.05 (compared to “Sham”), ^+^ *p* < 0.05, ^+++^ *p* < 0.001 (compared to “SNI”). (**g**) The number of the arm entries in the Y-maze, mean ± SEM, *n* = 20 (number of animals), ^+^ *p* < 0.05 (compared to “SNI”), ^#^ *p* < 0.05 (compared to “SNI + Syn”). SNI—spared nerve injury. Syn—synaptamide.

Allodynia is a condition in which pain is triggered by a stimulus that usually does not cause pain. A similar condition is a hyperalgesia, characterized by increased pain due to an irritant that usually causes pain. These symptoms are usually present in both peripheral neuropathy and pain disorders of central origin [28]. In the present work, we have studied thermal allodynia, as well as mechanical hyperalgesia in animals with spared nerve injury (SNI), after treatment with synaptamide and vehicle. The hot plate test showed a significantly longer period to the first paw withdrawal in synaptamide-treated animals with SNI compared to the vehicle-treated group with SNI. This tendency became noticeable from the 14th day (10.06 ± 2.12 vs. 24.48 ± 3.14, *p* < 0.001), and continued until the 28th day of observation (11.97 ± 2.43 vs. 21.09 ± 2.16, *p* < 0.001) (Figure 1a). Differences between the groups in the time of paw withdrawal from the cold plate became noticeable 21 days after the surgery. On day 21, the mean latency was 7.99 ± 1.56—“SNI” vs. 16.99 ± 2.64—“SNI + syn”, *p* < 0.001, on day 28 the latency was 8.31 ± 0.70—“SNI” vs. 14.85 ± 1.53—“SNI + syn”, *p* < 0.001 (Figure 1b). In the study of mechanical hyperalgesia, significant differences in the applied pressure causing the response of the animal were observed already on the 14th day after the surgery (294.38 ± 39.02—“SNI” vs. 175.65 ± 37.06—“SNI + syn”, *p* < 0.001) and continued the 28th day of observations (280.08 ± 53.83—“SNI” vs. 158.34 ± 35.95 “SNI + syn”, *p* < 0.001) (Figure 1c).

The influence of trauma and treatment on cognitive function, namely on long-term and working spatial memory indicators, was also studied. Long-term memory was examined in a novel object recognition test. The negative effect of neuropathic pain on long-term memory has been described in many works; therefore, the beneficial effect of drugs on this type of memory can serve as an indicator of the treatment effectiveness [16,29,30]. According to our results, the administration of synaptamide to mice prevented a recognition index decrease (40.86 ± 6.36 “SNI” vs. 65.04 ± 6.06—“SNI + syn”, *p* < 0.01) (Figure 1e). In the “SNI + Syn” group, the average time spent exploring a familiar object was significantly lower than that of a novel one (4.88 ± 1.03 familiar vs. 10.42 novel, *p* = 0.023), while in the SNI group, animals spent approximately the same amount of time on the study of the novel and familiar objects (12.71 ± 2.21—“SNI” vs. 15.11 ± 3.53—“SNI + Syn”) (Figure 1d). At the same time, at the familiarization session, the objects’ exploration rate by animals did not differ significantly. Since there are numerous data in the literature on working memory impairment in neuropathic pain [31,32,33], we investigated the spontaneous alternations rate in the Y-maze as an indicator of memory deficit in pain and treatment. In untreated mice with SNI, the index of working spatial memory was significantly lower than in synaptamide-treated animals (55.18 ± 2.95—“SNI” vs. 63.01 ± 2.81—“SNI + Syn”, *p* = 0.038) (Figure 1f). The influence of trauma and treatment on cognitive function, namely on long-term and working spatial memory indicators, was also studied. Long-term memory was examined in a novel object recognition test. The negative effect of neuropathic pain on long-term memory has been described in many works; therefore, the beneficial effect of drugs on this type of memory can serve as an indicator of the treatment effectiveness [16,29,30]. According to our results, the administration of synaptamide to mice prevented a recognition index decrease (40.86 ± 6.36—“SNI” vs. 65.04 ± 6.06—“SNI + syn”, *p* < 0.01) (Figure 1e). In the “SNI + Syn” group, the average time spent exploring a familiar object was significantly lower than that of a novel one (4.88 ± 1.03-familiar vs. 10.42-novel, *p* = 0.023), while in the SNI group, animals spent approximately the same amount of time on the study of the novel and familiar objects (12.71 ± 2.21 – ”SNI” vs. 15.11 ± 3.53—“SNI + Syn”) (Figure 1d). Since there are numerous data in the literature on working memory impairment in neuropathic pain [13,31,32], we investigated the spontaneous alternations rate in the Y-maze as an indicator of memory deficit in pain and treatment. In untreated mice with SNI, the index of working spatial memory was significantly lower than in synaptamide-treated animals (55.18 ± 2.95—“SNI” vs. 63.01 ± 2.81—“SNI + Syn”, *p* = 0.038) (Figure 1f). The number of entries into the Y-maze arms was used as an indicator of locomotor activity. Sciatic nerve injury did not significantly affect this indicator, while synaptamide administered to sham-operated mice increased the number of inputs compared to injured groups (*p* < 0.05) (Figure 1g).

### 2.2. Synaptic Plasticity in the Hippocampus upon Synaptamide Administration in Neuropathic Pain

**Figure 2:**
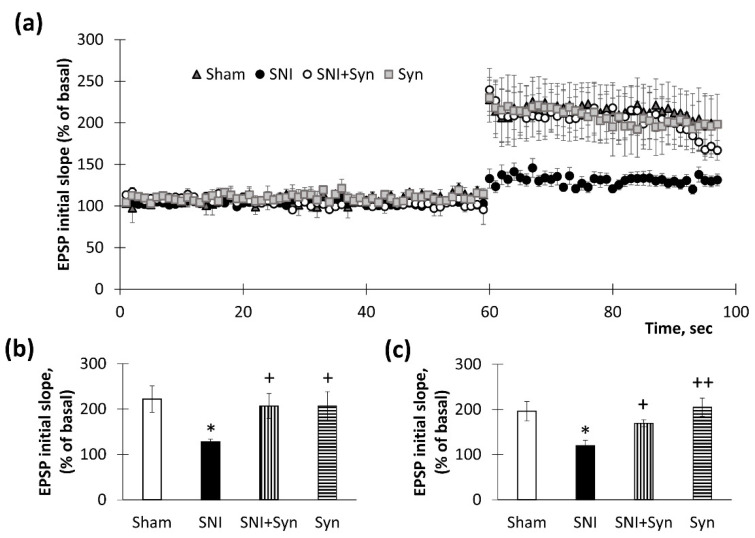
Synaptamide’s effect on SNI-induced long-term potentiation (LTP) inhibition. (**a**) Tetanus-induced LTP in the Schaffer collateral in SNI and synaptamide-treated mouse hippocampus slices. The information is presented as a mean percentage change in the slope of the population excitatory postsynaptic potential (EPSP). (**b**) The averaged initial slope, measured immediately after LTP, %, *n* = 8 (number of animals per group). (**c**) The averaged initial slope at 35–36 min after LTP, %, mean ± SEM, *n* = 8 (number slices per group). * *p* < 0.05 (compared to “Sham”), ^+^ *p* < 0.05, ^++^ *p* < 0.01 (compared to “SNI”), Kruskal–Wallis test followed by Dunn’s multiple comparisons tests, * *p* < 0.05 (compared to “Sham”), ^+^ *p* < 0.05, ^++^ *p* < 0.01 (compared to “SNI”).

The long-term potentiation in the CA1 area of acute hippocampal slices was measured to investigate hippocampal synaptic plasticity. Prior to tetanic stimulation, a steady baseline was recorded for 60 min. Long-term potentiation in the CA1 region was generated by tetanization of the Schaffer collateral–commissural pathway. The slope of the population excitatory postsynaptic potential (EPSP) is reported as a mean percentage change (Figure 2a). The normalized field EPSPs slopes in “SNI”, “SNI + Syn” and “Syn” groups amounted 132.94 ± 11.58% vs. 239.59 ± 25.72% (*p* < 0.05) and 230.03 ± 15.88 (*p* < 0.05) of baseline value immediately after tetanic stimulation (Figure 2b). In 35–36 min after tetanization EPSP slopes for “SNI”, “SNI + Syn”, and “Syn” were 119.77 ± 11.80% vs. 169.34 ± 7.97% (*p* < 0.05) and 204.20 ± 20.30 (*p* < 0.01) (Figure 2c).

### 2.3. Neuronal Tree Morphology upon Synaptamide Administration in Neuropathic Pain

The reorganization of the dendritic tree in the hippocampus is characteristic of many pathologies accompanied by chronic stress [33]. Atrophy of hippocampal neurons is also recorded in neuropathic pain models [34]. Changes in density, shape, and size of dendritic spines, are accompanied by changes in memory and learning and are observed in various neurological, mental, and neurodegenerative diseases. A change in the dendritic spines’ configuration has also been shown in neuropathic pain, which underlies the observed changes in synaptic plasticity [35,36].

**Figure 3:**
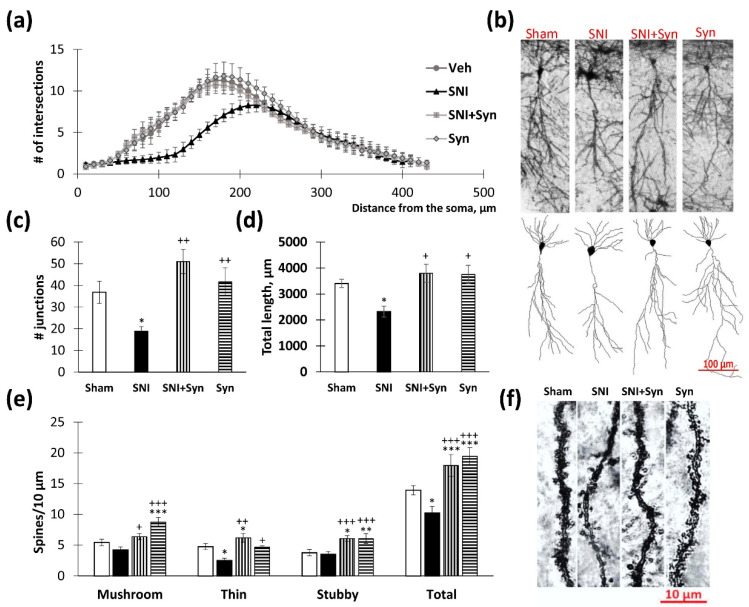
The results of dendrite Sholl analysis and spines density measurement. (**a**) The number of intersections along the apical dendritic trees at all distances from the soma in CA1 pyramidal neurons, Mean ± SEM, *n* = 5 (number of animals). (**b**) Representative images of CA1 pyramidal neurons in the contralateral dorsal hippocampus of mice with neuropathic pain and synaptamide treatment. (**c**) The number of junctions along the apical dendritic trees at all distances from the soma of CA1 pyramidal neurons, *n* = 5 (number of animals). (**d**) The total length of apical dendrites in CA1 pyramidal neurons, *n* = 5 (number of animals). (**e**) The density of dendritic spines in the apical dendrites of the CA1 pyramidal neurons. (**f**) The images of CA1 pyramidal neurons stained by the Golgi–Cox method, mean ± SEM, *n* = 10 (number of analyzed neurons per group), * *p* < 0.05, ** *p* < 0.01, *** *p* < 0.001 (compared to “Sham”), ^+^ *p* < 0.05, ^++^ *p* < 0.01, ^+++^ *p* < 0.001 (compared to “SNI”).

Using Sholl analysis, we identified signs of neuronal degeneration in the CA1 region of the hippocampus in neuropathic pain. At a distance of 60 to 200 μm from the soma in the “SNI” group, there is a significant decrease in the intersections’ number of pyramidal neuron dendrites compared to the “Sham” group (*p* < 0.05) (Figure 3a). In synaptamide-treated animals (“SNI + Syn” and “Syn”), the degree of branching did not differ significantly from the groups with sham-operated animals (Figure 3b). For a more detailed analysis of changes in the structure of dendrites, we compared the groups in terms of the “average number junctions” and “total length of dendrites”. Using the Kruskal–Wallis test, we found a significant reduction in the mean number of junctions in neuropathic pain (*p* = 0.037). At the same time, the indicators of the groups “SNI + Syn” and “Syn” did not differ significantly from the indicators of the “Sham” group (Figure 3c). Significant differences were shown between the “SNI” and “SNI + Syn” groups (20.04 ± 3.39 vs. 51.36 ± 5.57, *p* = 0.003, respectively). In addition, the Kruskal–Wallis test followed by the Dunn’s test showed that synaptamide also prevents an SNI-induced decrease in the total length of dendrites (2434.26 ± 210.03—“SNI” vs. 3795.62 ± 348.14—“SNI + Syn”, *p* = 0.02) (Figure 3d).

The two-way ANOVA revealed a significant effect of synaptamide on the density of mushroom spines in pyramidal neurons’ apical dendrites in the CA1 region (F(3, 40) = 21.215, *p* < 0.0001). In the group of synaptamide-treated sham-operated animals, the mushroom spines density was significantly higher than in vehicle-treated sham-operated animals and then in synaptamide-treated animals with SNI (5.42 ± 0.52—“Sham” vs. 8.74 ± 0.73—“Syn”, *p* < 0.001 and 6.35 ± 0.50—“SNI + Syn”, *p* < 0.08). Synaptamide prevented the decrease in the thin spines’ density observed in SNI (2.50 ± 0.39—“SNI” vs. 6.17 ± 0.69—“SNI + Syn”, *p* < 0.01). A 2-way ANOVA of stubby spines density revealed no effect of trauma and a significant effect of the treatment on this parameter (F(3, 40) = 16.59, *p* < 0.0001). In the synaptamide-treated groups “SNI + Syn” and “Syn” there was an increase in the stubby spines density, compared to the vehicle-treated groups “Syn” and “Sham” (3.77 ± 0.45 and 3.51 ± 0.45 vs. 6.05 ± 0.45—l number of spines revealed a significant effect size for both injury and synaptamide treatment (F(3, 40) = 4.14, *p* = 0.048 for SNI; F (3, 40) = 33.88, *p* < 0.001 for treatment) (Figure 3e,f). 

### 2.4. Microglial Activity within the Hippocampus in SNI and Synaptamide Treatment

Iba-1 (ionized calcium binding adapter molecule 1) is a marker expressed by all microglial cells. Iba-1 expression is increased in microglia activated when exposed to any damaging factors, for example, traumatic brain injury [37], inflammation [38], or ischemia [39]. We investigated the activity of hippocampal microglia in synaptamide-treated SNI mice.

**Figure 4:**
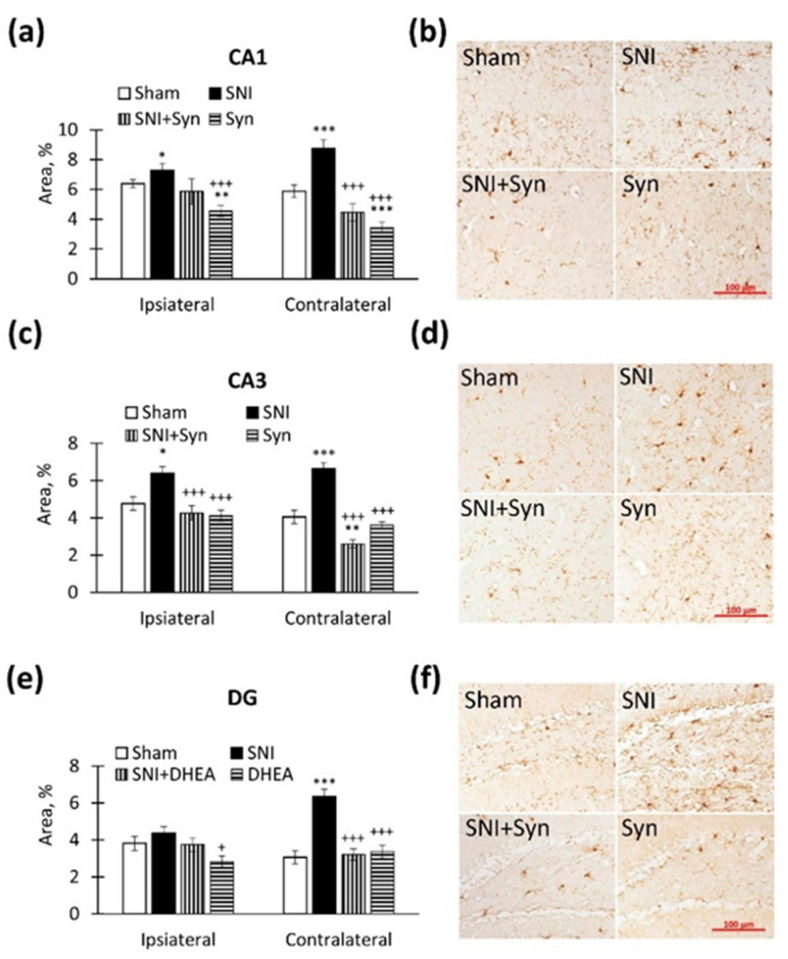
Iba-1 immunoreactivity in CA1, CA3 и DG hippocampal regions. (**a**) The percentage of Iba-1 immunopositive staining area in CA1 hippocampal region. (**b**) Representative images of Iba-1-positive immunostaining in CA1 hippocampal region. Scale bar — 100 µm. (**c**) The percentage of Iba-1 immunopositive staining area in CA3 hippocampal region. (**d**) Representative images of Iba-1-positive immunostaining in CA3 hippocampal region. Scale bar — 100 µm. (**e**) The percentage of Iba-1 immunopositive staining area in DG hippocampal region. (**f**) Representative images of Iba-1-positive immunostaining in DG hippocampal region. Scale bar — 100 µm. Two-way ANOVA with post hoc Tukey test, * *p* < 0.05, ** *p* < 0.01, *** *p* < 0.001; ^+^ *p* < 0.05, ^++^ *p* < 0.01, ^+++^ *p* < 0.001. *-compared to “Sham”, ^+^-compared to “SNI”.

We found that an immunopositive staining area increase in SNI within the CA1 region is observed both in the ipsi- (6.39 ± 0.28—“Sham” vs. 7.27 ± 0.49—“SNI”, *p* < 0.05) and in the contralateral hemisphere (5.87 ± 0.43—“Sham” vs. 8.76 ± 0.57—“SNI”, *p* < 0.001). Synaptamide down-regulated SNI-induced Iba-1 expression both in the ipsi- (7.27 ± 0.49 —“SNI” vs. 5.50 ± 0.43—“SNI + Syn”, *p* < 0.01) and in the contralateral hippocampus (8.76 ± 0.57—“SNI” vs. 4.48 ± 0.59—“SNI + Syn”, *p* < 0.001). Interestingly, synaptamide administered to sham-operated animals was able to reduce Iba-1 immunoreactivity compared to controls in both the ipsi- and the contralateral hippocampus. Two-way ANOVA showed a significant effect in the ipsilateral hippocampus for both injury (F(3, 40) = 7.12, *p* = 0.01) and treatment (F(3, 40) = 13.08, *p* = 0.001). A significant effect was also observed in the contralateral hippocampus: SNI (F(3, 40) = 32.04, *p* < 0.001), treatment: (F (3, 40) = 71.70, *p* < 0.001) (Figure 4a,b).

We observe a similar situation in the CA3 region (Ipsilateral: 4.76 ± 0.36—“Sham” vs. 6.39 ± 0.37—“SNI”, *p* < 0.05; Contralateral: 4.05 ± 0.36—“Sham” vs. 6.64 ± 0.33—“SNI”, *p* < 0.001). Synaptamide was also effective in Iba-1 down-regulation in SNI both in the ipsi- (6.39 ± 0.37—“SNI” vs. 4.26 ± 0.39—“SNI + Syn”, *p* < 0.001) and in the contralateral hippocampus (6.64 ± 0.33-”SNI” vs. 2.61 ± 0.22—“SNI + Syn”, *p* < 0.001). Interestingly—that in the “SNI + Syn” group the Iba-1 level was lower than in the “Sham” group (*p* < 0.01). Two-way ANOVA showed a significant effect in the ipsilateral hippocampus for both injury (F(3, 40) = 6.66, *p* = 0.013) and treatment (F(3, 40) = 16.45, *p* < 0.001). A significant effect was also observed in the contralateral hippocampus: SNI (F(3, 40) = 7.60, *p* = 0.008), treatment: (F (3, 40) = 60.81, *p* < 0.001) (Figure 4c,d). 

In DG we do not observe pronounced changes in the ipsilateral hemisphere after SNI, but in the contralateral hippocampus, the level of Iba1 immunoreactivity significantly upregulates (3.06 ± 0.35—“Sham” vs. 6.35 ± 0.39—“SNI”, *p* < 0.001). In the contralateral hippocampus, the Iba-1 level reversed to the “Sham” group in synaptamide treatment after SNI (6.35 ± 0.39—“SNI” vs. 3.21 ± 0.31—“SNI + Syn”, *p* < 0.001). Two-way ANOVA revealed a significant effect both for injury (F (3, 40) = 18.93, *p* < 0.001) and for treatment (F(3, 40) = 15.38, *p* < 0.001). It is noteworthy that in the ipsilateral hippocampus, synaptamide administered to sham-operated animals decreased the Iba-1 level below the “Sham” group, *p* < 0.05 (Figure 4e,f).

**Figure 5:**
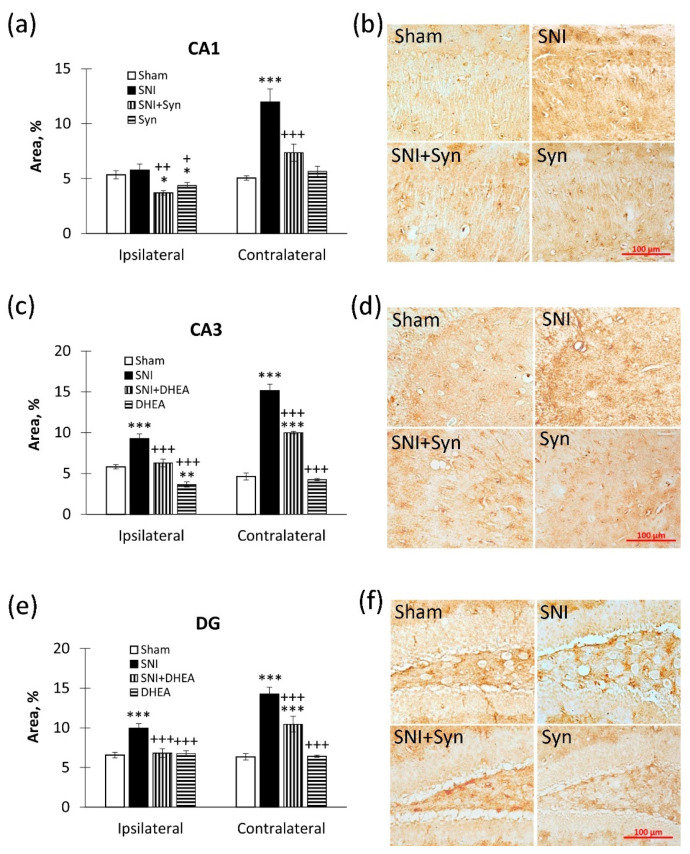
CD86 immunoreactivity in CA1, CA3 and dentate gyrus (DG) hippocampal regions. (**a**) The percentage of CD86 immunopositive staining area in CA1 hippocampal region. (**b**) Representative images of CD86-positive immunostaining in CA1 hippocampal region. Scale bar—100 µm. (**c**) The percentage of CD86 immunopositive staining area in CA3 hippocampal region. (**d**) Representative images of CD86-positive immunostaining in CA3 hippocampal region. Scale bar—100 µm. (**e**) The percentage of CD86 immunopositive staining area in DG hippocampal region. (**f**) Representative images of CD86-positive immunostaining in DG hippocampal region. Scale bar—100 µm. Two-way ANOVA with post hoc Tukey test, * *p* < 0.05, ** *p* < 0.01, *** *p* < 0.001; ^+^ *p* < 0.05, ^++^ *p* < 0.01, ^+++^ *p* < 0.001. *-compared to “Sham”, ^+^-compared to “SNI”.

As a second marker for microglial activity assessment in neuropathic pain and treatment, we used CD86, which is expressed in the cells of classically activated pro-inflammatory microglia [40]. We found no significant increase in CD86 immunoreactivity after SNI in the CA1 region of the ipsilateral hippocampus. Nevertheless, synaptamide reduces CD86 immunoreactivity compared to controls in both synaptamide-treated SNI animals and sham-operated mice (5.35 ± 0.37—“Sham” vs. 3.69 ± 0.21—“SNI + Syn”, *p* < 0.01 and 4.37 ± 0.27—“Syn”, *p* < 0.05). Two-way ANOVA showed a significant effect in the contralateral hippocampus for both injury (F(3, 40) = 4.09, *p* = 0.046) and treatment (F(3, 40) = 15.42, *p* < 0.001). In the contralateral hippocampus, we observe a significant increase in CD86 immunoreactivity in SNI compared to control (5.57 ± 0.54—“Sham” vs. 11.95 ± 1.20 —“SNI”, *p* < 0.001). Synaptamide down-regulated SNI-induced CD86 immunoreactivity (11.95 ± 1.20—“SNI” vs. 7.35 ± 0.78, *p* < 0.001). Two-way ANOVA showed a significant effect in the contralateral hippocampus for both injury (F(3, 40) = 50.75, *p* < 0.001) and treatment (F(3, 40) = 20.31, *p* < 0.001) (Figure 5a,b).

In the CA3 region of the ipsilateral hippocampus, synaptamide was able to reduce the SNI-mediated increase in CD86 immunoreactivity (9.26 ± 0.59—“SNI” and 6.28 ± 0.46 —“SNI + Syn”, *p* < 0.001). Two-way ANOVA showed a significant effect in the contralateral hippocampus for both injury (F(3, 40) = 65.08, *p* < 0.001) and treatment (F(3, 40) = 21.86, *p* < 0.001). In the contralateral hippocampus, the level of CD86 immunoreactivity in SNI was significantly increased compared to control (*p* < 0.001), but synaptamide reversed this indicator (15.12 ± 0.80—“SNI” vs. 9.99 ± 0.16—“SNI + Syn”, *p* < 0.001). Two-way ANOVA revealed a significant effect in the contralateral hippocampus for both injury (F(3, 40) = 380.71, *p* < 0.001) and treatment (F(3, 40) = 71.83, *p* < 0.001) (Figure 5c,d).

In the dentate gyrus, an increase in the CD86 immunopositive microglia level is observed both in the ipsilateral and in the contralateral hippocampus, compared to the control (*p* < 0.001). Synaptamide downregulated CD86 level both in the ipsi- (9.93 ± 0.60 – ”SNI” vs. 6.80 ± 0.54—“SNI + Syn”, *p* < 0.001) and contralateral hippocampus (14.23 ± 0.87—“SNI” vs. 10.45 ± 0.99—“SNI + Syn”, *p* < 0.001). Two-way ANOVA showed a significant effect in the contralateral hippocampus for both injury (F(3, 40) = 83.53, *p* < 0.001) and treatment (F(3, 40) = 14.40, *p* < 0.001) (Figure 5e,f).

### 2.5. Hippocampal Neurogenesis in SNI and Synaptamide Treatment

The study of hippocampal neurogenesis in neuropathic pain and synaptamide treatment was carried out using the immunohistochemical study of proliferating cell nuclear antigen (PCNA), a marker of proliferation and reparation, and doublecortin (DCX), a marker of newly formed neurons.

**Figure 6:**
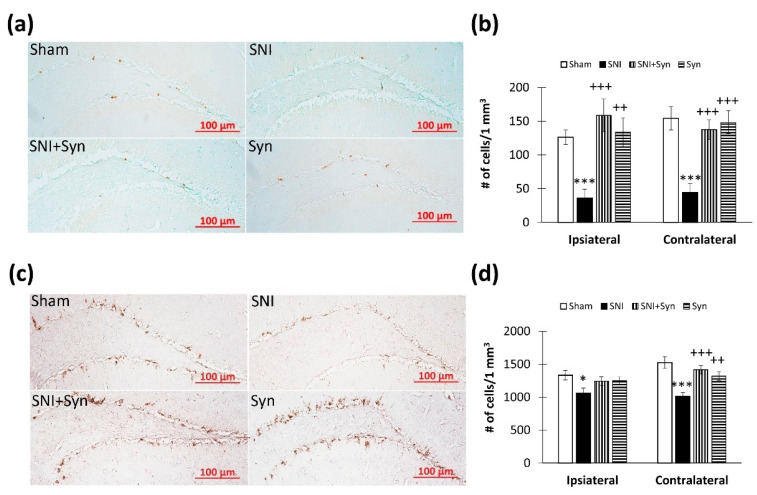
Hippocampal neurogenesis in SNI and synaptamide treatment. (**a**) Representative images of PCNA-positive cells in DG SGZ. (**b**) The number of PCNA-positive cells in DG SGZ. Scale bar—100 µm. (**c**) Representative images of DCX-positive cells in DG SGZ. (**d**) The percentage of DCX-immunopositive staining area in DG SGZ. Scale bar—100 µm. Two-way ANOVA with post hoc Tukey test, * *p* < 0.05, ** *p* < 0.01, *** *p* < 0.001; ^+^ *p* < 0.05, ^++^ *p* < 0.01, ^+++^*p* < 0.001. *-compared to Veh, ^+^-compared to LPS.

When studying the density of PCNA-positive neurons in the hippocampal dentate gyrus subgranular zone (DG SGZ) (Figure 6a), we found that in neuropathic pain, the number of cells significantly decreases in the ipsi- (126.39 ± 10.48—“Sham” vs. 35.72 ± 13.62—“SNI”, *p* < 0.001) and in the contralateral hippocampus (154.49 ± 17.40—“Sham” vs. 44.26 ± 13.16—“SNI”, *p* < 0.001). Synaptamide reversed SNI-mediated decrease in the number of PCNA-positive cells in the ipsi- (35.72 ± 13.62—“SNI” vs. 158.72 ± 24.26—“SNI + Syn”, *p* < 0.001) and the contralateral hippocampus (44.26 ± 13.16 – ”SNI” vs. 137.76 ± 14.64—“SNI + Syn”, *p* < 0.001). A 2-way ANOVA in the ipsilateral hippocampus revealed a significant effect for both injury (F(3, 40) = 16.62, *p* < 0.001) and treatment (F(3, 40) = 24.74, *p* < 0.001). A similar situation was observed in the contralateral hippocampus: (F(3, 40) = 35.53, *p* < 0.001)-SNI, (F (3, 40) = 16.90, *p* < 0.001)-treatment (Figure 6b).

There were insignificant changes in the number of DCX-positive neurons in the dentate gyrus subgranular zone of the ipsilateral hippocampus with SNI (1331.13 ± 72.23—“Sham” vs. 1060.06 ± 61.00—“SNI”, *p* < 0.05). At the same time, in the contralateral hippocampus, we observed a more pronounced increase in the number of DCX-positive cells compared to control (1525.07 ± 89.14—“Sham” vs. 1012.24 ± 61.00—“SNI”, *p* < 0.001). Synaptamide prevented SNI-mediated decrease in newly formed neurons (1012.24 ± 61.00— ”SNI "Vs. 1415.97 ± 64.96—“ SNI + Syn ", *p* < 0.001). Two-way ANOVA revealed a significant effect both for injury (F(3, 40) = 25.55, *p* < 0.001) and for treatment (F(3, 40) = 17.95, *p* < 0.001) (Figure 6c,d).

### 2.6. Neuropathic Pain and Treatment Alter the Hippocampal Level of Glutamate Receptors and PSD-95

**Figure 7:**
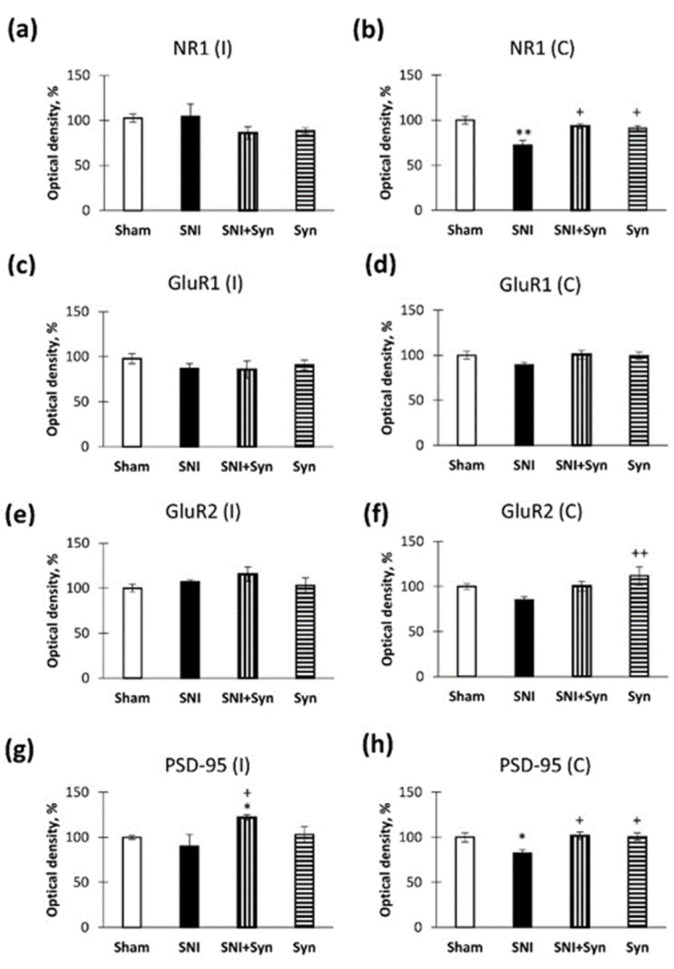
Neuropathic pain and treatment alter the hippocampal level of glutamate receptors and PSD-95. (**a**) Production of NMDAR1 (NR1) in ipsilateral hippocampus, optical density units, %. (**b**) Production of NMDAR1 (NR1) in contralateral hippocampus, optical density units, %. (**c**) Production of GluA1 in ipsilateral hippocampus, optical density units, %. (**d**) Production of GluA1 in contralateral hippocampus, optical density units, %. (**e**) Production of GluA2 in ipsilateral hippocampus, optical density units, %. (**f**) Production of GluA2 in contralateral hippocampus, optical density units, %. (**g**) Production of PSD-95 in ipsilateral hippocampus, optical density units, %. (**h**) Production of PSD-95 in contralateral hippocampus, optical density units, %., * *p* < 0.05, ** *p* < 0.01; ^+^ *p* < 0.05, ^++^ *p* < 0.01. *-compared to Veh, ^+^-compared to LPS.

We found that neuropathic pain alters the hippocampal levels of NMDA and AMPA receptors. The contralateral hippocampus was the most affected. Thus, in animals with SNI, the level of the NR1 subunit of NMDA receptors in the contralateral hippocampus decreased (100 ± 4.29%—“Sham” vs. 72.26 ± 5.38%—“SNI”, *p* < 0.01). There were no significant differences in the ipsilateral hippocampus (Figure 7a). However, upon syntamide treatment, the NR1 level remained at the level of the control group and significantly differed from the “SNI” group (72.26 ± 5.38%—“SNI” vs. 93.43 ± 2.73%—“SNI + Syn”, *p* < 0.05) (Figure 7b). However, neither trauma nor treatment affected the level of the NR2A subunit (data not shown). Moreover, there was no effect of SNI on the level of GluR1 and GluR2 AMPA subunits, although there was a downward trend (Figure 7c–f). When examining the level of the postsynaptic density protein PSD-95, we found the decrease in neuropathic pain in the contralateral hippocampus, but this effect was reversed by the synaptamide treatment (84.86 ± 3.60%—“SNI” vs. 101.94 ± 4.94%—“SNI + Syn”, *p* < 0.05) (Figure 7h). Interestingly, in the ipsilateral hippocampus, we observe an increase in the level of PSD-95 in the “SNI + Syn” group compared to the control (100 ± 2.16%—“Sham” vs. 121.87 ± 3.11%—“SNI + Syn”, *p* < 0.05) (Figure 7g).

## 3. Discussion

In this work, we studied the effect of docosahexaenoic acid derivative synaptamide, on neuropathic pain indicators, as well as on pain-induced changes in hippocampal plasticity. As expected, neuropathic pain manifested itself in a range of sensory symptoms, including thermal allodynia and mechanical hyperalgesia. The use of synaptamide improved sensory symptoms of neuropathic pain in mice. As in earlier studies [40], neuropathic pain disrupted the performance of hippocampus-dependent memory types: working and long-term. Behavioral studies showed that neuropathic pain interfered with the task of new objects’ recognizing in SNI, and synaptamide reversed these impairments. In addition, synaptamide prevented working memory impairment caused by neuropathic pain. These behavioral deteriorations are based on changes in the hippocampal neuronal and synaptic plasticity that we see in neuropathic pain. As you know, peripheral neurotrauma induces changes in the neurotransmitters’ release, as well as the expression of excitatory and inhibitory receptors, which affects synaptic transmission [7]. The impaired activity of glutamatergic transmission is considered as one of the neuropathic pain pathogenetic mechanisms [41]. In a study by Ultenius et al. (2006) [42], on sciatic nerve injury, increased phosphorylation of NR1 in the spinal cord ipsilateral dorsal horn in the rat was observed, which indicates a significant role of NR1 in the neuropathic pain pathogenesis. According to our experiment, SNI causes a reduction of NMDA NR1 subunits in the hippocampus. The NR1 subunit is important for the functioning of NMDA receptors, and NR1 phosphorylation is the main mechanism for regulating channel activity and its transfer to the neuronal surface [43]. Activation of protein kinase C enhances NMDAR activity and increases long-term potentiation (LTP). Considering the ability of synaptamide to enhance cAMP/PKC signaling, it is possible to explain the recovery of NR1 expression, impaired due to SNI, upon synaptamide administration. AMPA receptors are co-expressed with NMDAR at mature synapses, provide an initial response to glutamate at the synapse, and are involved in the neuropathic pain pathogenesis [44]. However, in most studies, the expression of this receptor subtype in the hippocampus remains unchanged. For example, in the work of Wang et al. (2015) there was no alteration of GluA1 or GluA2 expression within the hippocampus in partial sciatic nerve ligation (PSNL) model [45]. In the study by Goffer et al. (2013), there was no significant change in AMPA expression in SNI [46]. In our study, the subunits of the AMPA receptors GluR1 and GluR2 tend to decrease in the contralateral hippocampus during neuropathic pain; however, we did not find significant changes in the expression of AMPAR by ELISA. The absence of changes in the total level of AMPA receptors both in the previous ones [44,45] and in our study may be since only the surface density of receptors changes due to endocytosis, while the total number remains unchanged or changes slightly. However, studies show that pain stimuli in the spinal cord facilitate the trafficking of AMPA receptors to the cell surface [47,48]. In the anterior cingulate cortex, the level of receptors evenly increases on the cell surface and decreases in the cell cytoplasm [49]. In addition to the SNI-induced decrease in the level of NMDA receptors, a decrease in postsynaptic density 95 protein (PSD-95) was observed in the contralateral hippocampus. PSD-95 is usually co-expressed with NMDA receptors and it regulates NMDARs activity [50]. PSD-95 is one of the most abundant postsynaptic density proteins and regulates the synaptic localization of receptors, channels, and signaling molecules [51]. PSD-95 is considered an important regulator in the signaling complexes organization in NMDA receptors [52]. A decrease in PSD-95 at glutamate synapses in the DG molecular layer may negatively affect the flow of information to other hippocampal regions through granular cells and mossy fibers [53]. Synaptamide can recover the decreased level of NMDA receptors thereby contributing to the normalization of the PSD-95 level. Studies of PSD-95 indicate its possible role in the regulation of the dendritic spines’ structure. Overexpression of PSD-95 leads to an increase in the dendritic spines’ density, their stabilization, and the formation of synapses [54]. In our study, we observed an SNI-induced decrease in the density of thin spines on the apical dendrites of the CA1 pyramidal neurons. Thin spines, unlike mushroom ones, are more dynamic, since they are responsible for the formation of short-term memory. As the spine stabilizes, its head increases, which determines the transfer of information into long-term memory [55]. In this case, long-term potentiation (LTP) is directly related to the size and density of dendritic spines. LTP induction causes instant polymerization of actin filaments in the spine neck, contributing to a change in spine structure, and associated synaptic efficiency [56]. Interestingly, synaptamide administration not only reversed the decrease in the thin spines’ density, but also increased the number of mushroom and stubby spines compared to the control. The role of stubby dendritic spines is still poorly understood, and they are considered formed, mainly due to the disappearance of mushroom spines. Recent studies have shown that stubby and mushroom spines have similar mean protein copy numbers and topology [57], which also suggests that stubby spine density is related to changes in mushroom density.

The injury of peripheral nerves provoked not only a dendritic spines density decrease but also degradation of the apical dendrites in CA1-pyramidal neurons. Previous studies show that maladaptive changes in the dendritic tree within the hippocampus lead to dysregulation of synaptic plasticity [58], which likely provokes the development of cognitive and emotional symptoms of neuropathic pain. For synaptic integration, voltage-gated channels in pyramidal dendrites are of great importance [59]. Morphological changes and degeneration of pyramidal neurons are associated with the microglia activation and the production of pro-inflammatory factors [35,60,61]. The present study demonstrates the activation of microglia predominantly in the contralateral hippocampus due to SNI. The administration of synaptamide effectively suppressed SNI-induced microglial activation. At the same time, synaptamide treatment caused the recovery of the length and branching in the CA1 pyramidal neurons’ dendrites and improvement of hippocampal neurogenesis, impaired due to SNI. It can be assumed that such a beneficial effect on dendrite morphology and neurogenesis is due to the anti-inflammatory activity of synaptamide. The anti-inflammatory activity of synaptamide has been demonstrated previously in in vitro [26,62] and in vivo [63] models, and is considered to be associated with binding to the GPR110 receptor, leading to activation of the cAMP/PKA signaling pathway and NF-κB inhibition. Activation of the cAMP/PKA signaling pathway is realized through the cyclic adenosine monophosphate (cAMP) accumulation in cells, followed by cAMP-dependent phosphorylation of the protein kinase A (PKA) enzyme and suppression of NF-kB activity. Since NF-κB induces the expression of various pro-inflammatory genes, its suppression leads to a decrease in the inflammatory response, which is manifested by a decrease in the production of the pro-inflammatory cytokines and an increase in the anti-inflammatory factors production by microglial cells [63]. Proinflammatory cytokines derived from microglia induce impaired neurogenesis, thereby provoking memory and learning failure [64]. The involvement of the lateral entorhinal cortex (lEC) neurons in the sensory information transmission into the hippocampus, the axons of which terminate on the DG dendrites [9], may explain the significant activation of microglia in this region, as well as neurogenesis impairment, and the disturbances of nonspatial processing. The connection between the medial entorhinal cortex (mEC) and the hippocampus is known to play an important role in working memory. In mEC lesions, there is an extensive deficit in the spatial coding of CA1 pyramidal neurons, accompanied by less informative spatial firing patterns [65]. The studies show the involvement of the CA1 region in the long-term recognition memory implementation [66]. Thus, increased neuronal activity in the CA1 region can provoke microglial activation [67,68] with subsequent degeneration of the neuronal tree due to hyperactivation and excitotoxicity [69,70,71]. The projections from layer II of the entorhinal cortex onto the neurons of the CA3 region, along with projections in the CA1 region, take part in the so-called temporoammonic pathway of sensory information entering the hippocampus. An increase in the microglial activity within the CA3 region seems to be a consequence of neuronal activation [68]. According to our data, an increase in the CD86-immunoreactivity (a marker of proinflammatory M1-type of microglia), is observed in CA1, CA3, and DG. Factors produced by M1 microglia are involved in neuropathic pain pathogenesis [72,73]. For example, an increase in the proinflammatory cytokine IL-1β level in the hippocampus controls the mechanical allodynia development [74]. Another proinflammatory brain cytokine, TNF-α, is also associated with a pain hyper response in sciatic nerve ligation in rats [75]. We assume that synaptamide inhibits the hippocampal neurons’ hyperactivation and prevents the dendritic tree structure disruption, by suppressing the microglial activity and the production of the proinflammatory cytokines. Suppression of neuroinflammation processes leads to the stabilization of the neurotransmitters release, expression of excitatory and inhibitory receptors [76], as well as the dendritic spines’ density and configuration [77]. Together, these properties have a beneficial effect on the hippocampal functional state, including synaptic transmission and hippocampus-dependent cognitive processes.

## 4. Materials and Methods

### 4.1. Animals

We used 3-month-old male mice in the study. Mice were raised in the National Scientific Center of Marine Biology, Far Eastern Branch of the Russian Academy of Sciences, Vladivostok, Russia. The cage contained 3–4 mice with ad lib access to food and water. Animals were housed on a 12-h light/dark cycle at 23 ± 2 °C and 55 ± 15% humidity. The experimental procedures were approved by the Animal Ethics Committee at the National Scientific Center of Marine Biology, Far Eastern Branch, Russian Academy of Sciences (No. 3/2021), according to the Laboratory Animal Welfare guidelines and the European Communities Council Directive 2010/63/EU.

### 4.2. Surgery and Treatment

The spared nerve injury model (SNI) [78] was used to induce neuropathic pain. A rodent anesthetic vaporizer (VetFloTM, Kent Scientific Corporation, Torrington, CT, USA) was applied to anesthetize the mice using isoflurane. The right sciatic nerve was exposed after the animal had been anesthetized, and two of the three sciatic nerve terminal branches (the tibial and common peroneal nerves) were tightly ligated (4–0 silk suture; Ethicon, Irvine, CA, USA). The ligatures were tightened until the limb began to twitch slightly. Distal to the ligature, the ligated branches were transected, and 2 mm of each distal nerve stump was excised. The sciatic nerve and its branches were exposed in the "Sham" group, but they were neither ligated nor transected. The muscles and skin of each animal were sutured individually with a 4–0 silk suture (Ethicon, Irvine, CA, USA).

**Figure 8:**
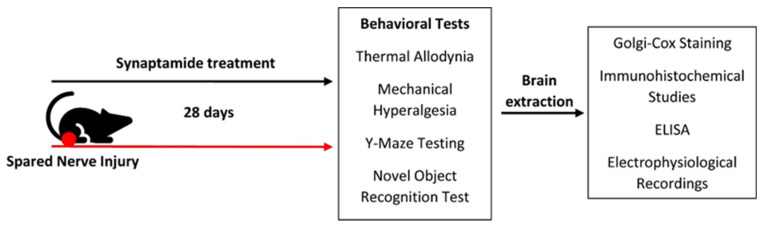
Experimental design. The thermal allodynia and mechanical hyperalgesia were measured weekly for two consecutive days. Memory tests were performed at days 27 and 28 after the surgery. Extraction of the brain for subsequent histological, immunohistochemical, biochemical, and electrophysiological studies was carried out on the 29th day after the surgery.

Synaptamide was injected subcutaneously (s.q.) in a dose of 10 mg/kg. The mice (n = 80) were divided into the following groups: “Sham” (n = 20)-water-injected sham-operated mice; “SNI” (n = 20)-water-injected mice with SNI; "SNI + Syn" (n = 20)-synaptamide-injected mice with SNI; “Syn” (n = 20)-synaptamide-injected sham-operated mice. The synaptamide injections were administered for 28 consecutive days. The first injection was carried out in a day of surgery. As a control, animals were treated with water in the same volume (100 μL). The synaptamide emulsion was prepared by shaking synaptamide with water to give a final concentration of 25 mg/mL, using a multi-vortex shaker (V-32, Biosan, Riga, Latvia). To increase the stability of the emulsion when gradually dissolving, ethanol was added in a low concentration. The amount of ethanol was 1.5% of the volume injected. A similar amount of ethanol was added to the water administered to the control animals. A brief design of the experiment is shown in Figure 8.

### 4.3. Synaptamide Preparation

Synaptamide was derived from the by-products of Bering Sea salmon. The PUFA concentrate was made using the technique reported before [79]. Ethanolamines were first made by converting a polyunsaturated fatty acid (PUFA) concentrate into ethyl esters and then treating them with ethanolamine. At least 48 h were spent incubating with ethanolamine at 70 °C. Then, using a Shimadzu LC-8A chromatograph (Shimadzu, Japan) with UV/VIS SPD-20A, HPLC of PUFA ethanolamides was performed (205 nm). Supelco Discovery HS C-18 preparative reverse phase column was used to separate ethanolamides (Bellefonte, PA, USA). The following parameters were used: particle size of 10 μm, inner diameter of 250 mm, and length of 50 mm. We used ethanol/water (70:30, *v*/*v*) for isocratic elution. The elution rate was 50 m per min. Fractions containing the resultant N-acylethanolamines were collected, evaporated in vacuo, and GC and GC–MS analyses were performed. At room temperature, the resultant synaptamide was a light-yellow oily liquid with a slight odor. Synaptamide has a purity of 99.4 percent.

The conversion to trimethylsilyl derivatives (TMS-NAE) was used to identify the composition of the ethanolamides [80]. 50 L of N, O-bis (trimethylsilyl) trifluoroacetamide (BSTFA) was mixed with 1 mg of fatty acid ethanolamides and heated to 60 °C under argon for 1 h. Then 1 mL of hexane was added, and 1 μL of each silylated fraction was injected into the GC apparatus to measure the ethanolamides composition. The chromatograph was a Shimadzu GC-2010 plus with a Supelco SLB TM–5 ms capillary column 30 m 0.25 mm inner (Sigma-Aldrich, Bellefonte, PA, USA) and a flame ionization detector (Shimadzu, Kyoto, Japan). To separate the components of the mixture, the following conditions were used: (1) a starting temperature of 180 °C; (2) a heating rate of 2 °C/min to 260 °C; and (3) the temperature was maintained for 35 min. The temperatures of the injector and detector were the same, at 260 °C. GC–MS was employed to identify the TMS–NAE structures. At 70 eV, a Shimadzu TQ-8040 instrument (Shimadzu, Kyoto, Japan) with a Supelco SLB TM−5 ms column (Sigma-Aldrich, Bellefonte, PA, USA) was used to record electronic impact spectra. The same temperature settings as in gas chromatography were applied.

### 4.4. Behavioral Tests

All behavioral tests were carried out throughout the day/night cycle’s light phase, from 7:00 to 19:00. To reduce olfactory signals, the test apparatus was carefully cleaned with 10% ethanol after each animal. Mice were placed in the test apparatus for 10 min daily for 3 days before the day of testing to prevent stress associated with the new environment. The mice were left in their home cages in the test room for two hours on the day of the test. Thermal allodynia was assessed on a weekly basis. In the 28 days following the procedure, memory tests were carried out.

### 4.5. Thermal Allodynia

A cold/hot plate analgesiometer was used to measure thermal allodynia (Columbus Instruments, Columbus, OH, USA). The experiments were performed on a 30 × 30 cm metal plate in a chamber with 30 cm high acrylic walls. The cold plate had a temperature of +4 °C, while the hot plate had a temperature of +48 °C, and the testing time was 60 s. Mice were placed on the plate, and the time when the injured hind paw was removed off the plate for the first time was recorded. This test was carried out twice a week for two consecutive days.

### 4.6. Mechanical Hyperalgesia

A mouse pincher analgesia meter (Bioseb, Pinellas Park, FL, USA) was used to measure mechanical hyperalgesia. The mice were gently held and increasing pressure was applied on the dorsal surface of the injured hind paw until the toes flexed. To avoid tissue injury, a 400-g threshold has been determined. This test was carried out twice a week for two consecutive days.

### 4.7. Y-Maze Testing

The Y-maze spontaneous alternation test was used to assess mice working memory. A Y-shaped acrylic glass labyrinth with three identical arms was used in the experiment (30 × 10 × 20 cm). The mouse was placed in the maze’s middle and given 5 min to explore freely. To determine the spontaneous alternation rate, the sequence of the entries was recorded. When the animal’s four paws were inside the arm, it was thought that the entrance had been made. Before the animals were sacrificed, this test was performed once every four weeks. The spontaneous alternation rate was calculated using the following formula:

Ks = R/A(1)

where Ks—the spontaneous alternation rate, R—the number of consecutive entries into the 3 nonrepeating arms, A—the total number of possible alternations.

### 4.8. Novel Object Recognition Test

The novel object recognition test was performed as described earlier in [81], Bevins and Besheer (2006). The habituation phase was carried out the day before the familiarization session. Each animal was placed in a chamber without objects and allowed to explore the space for 10 min. Each mouse was placed in a chamber containing two identical plastic objects on the left and right sides of the arena for 10 min during the familiarization phase. The animal was then returned to its original cage for a retention period of 24 h (for long-term memory testing). Each mouse was placed in a test arena where one of the objects was swapped with a novel one for testing purposes. Both items were positioned at the same distance from the animal. A recording device was put over the testing setup and was used to continually capture mouse activity. The placement of the animal’s nose at not more than 2 cm from the object was used to determine the animal’s interest in the object. The time spent investigating a new object was divided by the total time spent examining both objects to get the discrimination index. The objects and the arena were carefully cleaned with 10% ethanol in between experiments. This test was performed four weeks following the surgery before the animals were sacrificed.

### 4.9. Golgi–Cox Staining

A rodent anesthetic vaporizer (VetFloTM, Kent Scientific Corporation, Torrington, CT, USA) was used to anesthetize the mice using isoflurane. Brains were promptly taken from the skulls of anesthetized mice, washed with 0.1 M PBS (+4 °C), and sliced into two hemispheres. According to the manufacturer’s instructions, the material was stained with the FD Rapid GolgiStain^TM^ kit (FD NeuroTechnologies, Ellicott City, MD, USA). A cryomicrotome was used to make 100-m thick slices (HM 550; Thermo Scientific, Waltham, MA, USA). Slices were mounted on gelatin-coated slides, stained, dehydrated, and coverslipped with VectaMount^TM^ mounting medium (H-5000; Vector Laboratories, Burlingame, CA, USA).

### 4.10. Sholl Analysis

We used sagittal slices from the contralateral dorsal hippocampus to assess dendritic tree morphology. The impact of neuropathic pain and synaptamide therapy on the dendritic tree morphology of hippocampal CA1 pyramidal neurons was studied using a Sholl analysis [82]. For image processing and morphometrical studies, ImageJ software (NIH, USA) was utilized. Pictures for each individual neuron were transformed to 8-bit color images for dendritic tracing. As previously stated, [83], dendrites were traced using the NeuronJ plugin (NeuronJ: An ImageJ Plugin for Neurite Tracing and Analysis. Available online: http://www.imagescience.org/meijering/software/neuronj/ (accessed on 1 August 2021)). The Sholl analysis plugin (Sholl Analysis. Available online: https://imagej.net/plugins/sholl-analysis (accessed on 1 August 2021)) was used to do the analysis in ImageJ. The single animals were chosen as the analytical unit (5 animals per group). Moreover, 3–4 well-stained neurons were chosen from each animal for evaluation. 

### 4.11. Immunohistochemical Studies

On the 28th day following surgery, the brains were taken from the skull for immunohistochemistry tests. A rodent anesthetic vaporizer (VetFloTM, Kent Scientific Corporation, Torrington, CT, USA) was used to anesthetize the mice using isoflurane. The animals were transcardially perfused with 5 mL of pH 7.2 PBS (at 4 °C). The brain was then rapidly removed from the skull and immersed in 4% paraformaldehyde for 12 h. The tissue samples were then embedded in paraffin blocks after being rinsed in PBS (pH 7.2). A Leica rotary microtome was used to cut 10-m thick coronal slices (RM 2245). The following stages were included in the immunohistochemistry approach utilized in the research: (1) antigen retrieval in 10 mM citrate buffer, pH 6, at 80 °C for 20 min (only for Iba-1 and CD86 immunostaining). (2) Endogenous peroxidase activity blocking in 0.3 percent H_2_O_2_ solution for 5 min. (3) Nonspecific antibody binding blocking in 5 percent BSA in PBS for 1 h. (4) Primary antibody treatment (4 °C, 24 h). (5) Secondary antibodies labeled with horseradish peroxidase: PI1000 (anti-rabbit), 1:100 (Vector Laboratories, Burlingame, CA, USA). (6) ImmPACTTM DAB peroxidase substrate chromogen (SK-4105; Vector Laboratories); and (7) washing with 0.1 M PBS (pH 7.2), dehydrating, and mounting in VectaMount permanent mounting medium (Vector Laboratories, Burlingame, CA, USA) (H-5000; Vector Laboratories). Anti-Iba-1 rabbit polyclonal antibodies (1:500; ab108539), anti-CD86 rabbit monoclonal antibodies (1:1000; ab53004), anti-PCNA rabbit monoclonal antibodies (1:1000; ab92552), and anti-doublecortin rabbit polyclonal antibodies (1:1000; ab18723) were used for immunostaining (all from Abcam, Cambridge, MA, USA). Every method was used to quantify Iba-1, CD86, PCNA, and doublecortin-immunopositive cells. A Zeiss Axio Imager microscope with an AxioCam 503 camera and AxioVision (Zeiss, Germany) software was used to evaluate the images. ImageJ was used to process and analyze the images (NIH, Bethesda, MD, USA). The following methods were used to process each micrograph: conversion to black and white (8-bit picture); background subtraction (rolling ball radius = 50); contrast enhancement (+30 units); and binarization. The appropriate area was picked, and the percent-colored area was computed to quantify the extent of marker staining. The quantitative data of all photos of the same marker collected from one animal were averaged for statistical analysis.

### 4.12. ELISA

The concentration of NR1, GluA1, GluA2, and PSD-95 in the hippocampus was measured using an ELISA (enzyme-linked immunosorbent assay). A unique cohort of mice was used in this study (5 per group). The animals were sedated with isoflurane using a rodent anesthetic vaporizer (VetFloTM, Kent Scientific Corporation, Torrington, CT, USA). The hippocampi were rapidly removed, frozen in liquid nitrogen, and preserved at a temperature of −70 °C. The hippocampi were homogenized in a homogenization solution containing 100 mM Tris, pH 7.4, 150 mM NaCl, 1 mM EGTA, and 1 mM EDTA, 1% Triton X-100, 0.5% sodium deoxycholate, and a protease inhibitors cocktail (cOmplete™, Sigma-Aldrich, Bellefonte, PA., USA), kept on ice for 15 min, centrifuged (16,000× *g*, 30 min, +4 °C) and the supernatants were collected. The materials (supernatants of cells or tissue lysates) were diluted with bicarbonate-carbonate coating buffer (100 mM, 3.03 g Na_2_CO_3_, 6.0 g NaHCO_3_, 1000 mL distilled water, pH 9.6) to reach a concentration of 20 g/mL to identify NMDAR1, GluA1, GluA2, and PSD-95 antigens. The samples were then placed in each well of a PVC microtiter plate (M4561-40EA, Greiner, Austria) and incubated overnight at 4 °C. The coating solution was then withdrawn, and the plate was rinsed three times with 200 µL PBS in the wells. Moreover, 5% non-fat dry milk (M7409-1BTL, Sigma-Aldrich, St. Louis, MO, USA) was used to block the remaining protein-binding sites in the coated wells (2 h at room temperature). After washing 100 µL of diluted primary antibody was added to each well. Rabbit polyclonal anti-NMDAR1 (1:1000, ab52177, Abcam, Cambridge, UK), rabbit polyclonal anti-GlyA1 (1:1000, MA5-32344, Thermo Fisher Scientific, Waltham, MA, USA), rabbit polyclonal anti-GlyA2 (1:1000, 32-0300, Thermo Fisher Scientific, Waltham, MA, USA), rabbit polyclonal anti-PSD-95 (1:1000 (1:1000, 700902, Thermo Fisher Scientific, Waltham, MA, USA) primary antibody were used. The plate was covered with adhesive plastic and left to incubate at room temperature for 2 h. After washing, 100 µL of peroxidase secondary antibody (1:500, PI-1000-1, Vector laboratories, San Francisco, CA, USA) was added to each well, and incubated for 2 h at room temperature. After washing, 50 µL of TMB (3,3′,5,5′-tetramethylbenzidine, SK-4400, Vector laboratories, San Francisco, CA, USA) was added to each well, and incubated at room temperature for 30 min until color appeared. After the color had grown sufficiently, the wells were filled with 50 µL of stop solution (1N hydrochloric acid). At a wavelength of 450 nm, the absorbance was measured in an iMark plate spectrophotometer (Bio-Rad, Hercules, CA, USA). The measurements of each sample were averaged after they were tested twice. The protein concentration was measured using a BCA Protein Assay Kit (Pierce, Rockford, IL, USA).

### 4.13. Electrophysiological Recordings 

Mice were profoundly sedated with isoflurane (Laboratorios Karizoo, S.A., Barcelona, Spain), decapitated, and the brains were promptly removed and placed to an ice-cold aCSF containing 119 mM NaCl, 2.5 mM KCl, 2 mM MgCl_2_, 0,25 mM CaCl_2_, 26 mM NaHCO_3_, 1 mM NaH_2_PO_4_, 10 mM D-glucose, pH 7.4, oxygenated with carbogen 95% O_2_, 5% CO_2_. Using a vibratome, parasagittal slices with a thickness of 350 μm were produced from the hippocampus. Within 1 h, at 33 °C, the slices were allowed to recover. The recordings were made in a submersion-recording chamber that was perfused with aCSF at a temperature of 30 ± 0.5 °C and a flow rate of 2 mL/min. We used a nylon mesh fastened on a U-shaped platinum wire during aCSF perfusing to secure the segment in the recording chamber. An upright microscope was used to examine acute hippocampus slices (Olympus BX50, Olympus Corporation, Shinjuku City, Tokyo, Japan). The following recording extracellular electrode characteristics were used: an outer diameter of 1.5 mm, a length of 10 cm, and borosilicate glass (World Precision Instruments, Sarasota, FL, USA) (World Precision Instruments, Sarasota, FL, USA). Pt-Ir wire insulated by Teflon (75 m diameter, including Teflon covering) served as the monopolar stimulating electrode. The stimuli were triggered with an isolating stimulator and National Instruments LabVIEW 2019 software (10 s duration, Master8) (Constant Current Stimulus Isolator WPI). With a sampling rate of 15 Hz, an intracellular amplifier in bridge circuit mode (Axoclamp 2B, Axon Instruments, Berkeley, CA, USA) was applied. The signal was digitized (National Instruments, PCI 6154), analyzed, and filtered with LabVIEW 2019 software (National Instruments, Austin, TX, USA).

Between the CA2 and CA1 areas, the stimulating electrode was inserted into the Schaffer collateral fiber tract. To record an extracellular population excitatory postsynaptic potential (EPSP), an electrode was placed in the stratum radiatum subfield of the CA1 area at a distance of no more than 1500 μm, but no less than 300 μm from the stimulating electrode to avoid direct stimulation of cells near the recording sites. An extrasynaptic potential was noticed during 0.5 mA stimulation to see if the slice was acceptable for recording, and the typical graph of input/output stimulation currents (IO) was recorded. To stabilize the responses, we used a stimulation with a frequency of 1 Hz and a current of 0.4 mA for 30 min. The magnitude of the testing stimulus for long-term post-tetanic potentiation was 70% of the maximal extrasynaptic potential amplitude. Long-term potentiation (LTP) was achieved by stimulating the brain at 100 Hz for 1 s.

### 4.14. Statistical Analysis

All results are provided as means with standard error of the mean (means ± SEM). The Shapiro–Wilk test was used to determine if the data were normally distributed. We utilized one-way ANOVA followed by a post hoc Tukey multiple comparison test or Kruskal–Wallis test followed by Dunn’s multiple comparison testing in behavioral and electrophysiological recording. Histology, immunohistochemistry, and ELISA data were analyzed statistically using a two-way ANOVA followed by a post hoc Tukey multiple comparison test. The significance level was set at *p* < 0.05. Microsoft Excel software (Microsoft, Redmond, WA, USA) and GraphPad Prism 4 were used to conduct all statistical tests (GraphPad Software, San Diego, CA, USA). 

## Data Availability

The datasets used and analyzed during the current study are available from the corresponding author upon reasonable request.
